# Polyurethane Foams: Past, Present, and Future

**DOI:** 10.3390/ma11101841

**Published:** 2018-09-27

**Authors:** Nuno V. Gama, Artur Ferreira, Ana Barros-Timmons

**Affiliations:** 1 CICECO—Aveiro Institute of Materials and Department of Chemistry, University of Aveiro–Campus Santiago, 3810-193 Aveiro, Portugal; nuno.gama@ua.pt (N.V.G.); artur.ferreira@ua.pt (A.F.); 2 Escola Superior de Tecnologia e Gestão de Águeda-Rua Comandante Pinho e Freitas, No. 28, 3750-127 Águeda, Portugal

**Keywords:** polyurethane foams, sustainability, enhancement of properties, new processing methodologies

## Abstract

Polymeric foams can be found virtually everywhere due to their advantageous properties compared with counterparts materials. Possibly the most important class of polymeric foams are polyurethane foams (PUFs), as their low density and thermal conductivity combined with their interesting mechanical properties make them excellent thermal and sound insulators, as well as structural and comfort materials. Despite the broad range of applications, the production of PUFs is still highly petroleum-dependent, so this industry must adapt to ever more strict regulations and rigorous consumers. In that sense, the well-established raw materials and process technologies can face a turning point in the near future, due to the need of using renewable raw materials and new process technologies, such as three-dimensional (3D) printing. In this work, the fundamental aspects of the production of PUFs are reviewed, the new challenges that the PUFs industry are expected to confront regarding process methodologies in the near future are outlined, and some alternatives are also presented. Then, the strategies for the improvement of PUFs sustainability, including recycling, and the enhancement of their properties are discussed.

## 1. Polymeric Foams

Materials such as plastic foams, foamed plastics, cellular plastics, or polymeric foams are materials that consist of a solid phase and a gas phase [1]. Polymer foams can be rigid, flexible, or elastomeric, and can be produced from a wide range of polymers, such as polyurethane (PU), polystyrene (PS), polyisocyanurate (PIR), polyethylene (PE), polypropylene (PP), poly(ethylene-vinyl acetate) (EVA), nitrile rubber (NBR), poly(vinyl chloride) (PVC), or other polyolefins, being the world foam production dominated by PU foams (PUFs), followed by PS and PVC foams [2,3]. The global market for polymeric foams was worth more than $100 billion in 2015, with sales of more than 22 million tons and a consumption of 25.3 million tons is expected by 2019 [4,5]. Being lightweight materials whose properties can be easily tuned, polymeric foams are the first choice for a wide range of applications such as: packaging, automotive, electronics, furnishing, footwear, aerospace, toys, food contact, or construction materials [3,6]. PUFs [7] are commonly used in comfort applications or as thermal and sound insulation materials, PS foams [8] are commonly used as food packaging, thermal, and sound insulation materials, and PVC foams [9] are commonly used as transport and construction materials.

## 2. Polyurethane Foams

The first urethane was synthesized in 1849 by Wurtz [10]. Afterwards, in 1937, Otto Bayer synthesized PUs from the reaction between a polyester diol and a diisocyanate [10,11,12,13]. Indeed, this was a major breakthrough at the time, as it consisted of a new class of polymerization reaction called polyaddition, which is also known as step polymerization [10,12]. Nevertheless, at first, this polymer was considered useless [11].

**Scheme 1:**
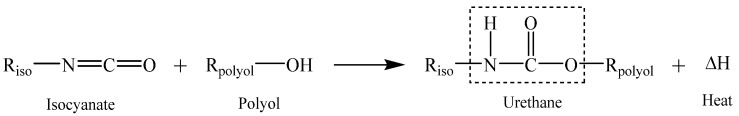
Reaction scheme of urethane production [10].

PUs are polymers that are formed by the reaction between the OH (hydroxyl) groups of a polyol with the NCO (isocyanate functional group) groups of an isocyanate, and the name is associated with the resulting urethane linkage [7,11,12,14]. This reaction is exothermic, and leads to the production of urethane groups as described before and illustrated in Scheme 1 [7,10]. 

Where *R_iso_* is derived from the isocyanate monomer, while *R_polyol_* is derived from the polyol component.

**Figure 1:**
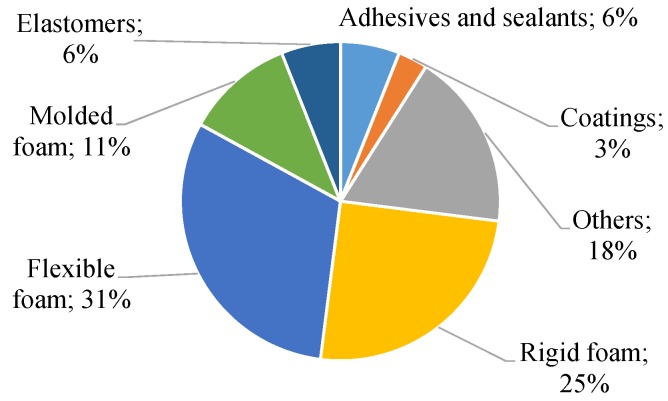
Global consumption of polyurethane (PU) in 2016.

Nowadays, PUs are used as everyday life products, being one of the most important class of polymers that keep changing the quality of the human life [10]. The worldwide consumption of PU was estimated at 60.5 billion USD in 2017, and it was predicted to be over 79 billion USD by 2021 [15]. In 2016, it represented nearly 9% of the global consumption of plastics [16]. Moreover, as illustrated in Figure 1, the principal consumption of PUs is in the form of foams [17]. 

Among PU consumption, PUFs correspond to 67% of global PU consumption. Furthermore, since the technology to produce is so well-established, this type of foams corresponds to half of the whole polymeric foam’s market [11]. The main types of PUFs are the flexible foams and rigid foams; nevertheless, other classifications can be attributed to PUFs, such as flexible PU slabs, flexible molded foams, reaction injection molding (RIM), carpet backing, or two-component formulations, etc. [7,11].

**Scheme 2:**
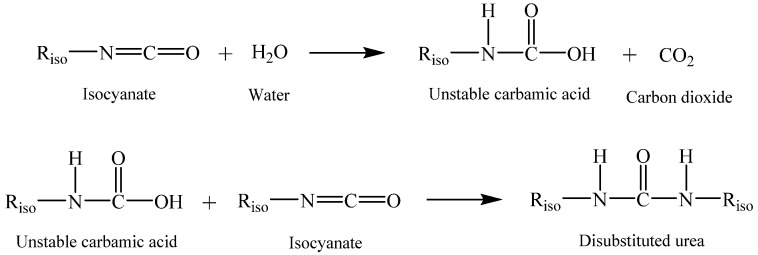
Reaction scheme of the isocyanate with water [10].

PUFs are expanded materials, and their structure results from the combination of several processes. First, the reactants are mixed together, next, a polymerization (reaction between isocyanate and polyol—see Scheme 1) and expansion (reaction between isocyanate and water—see Scheme 2) take place almost simultaneously [7].

PUFs correspond to 50% of global PU consumption, since the technology to produce them is so well-established when compared with foams derived from other polymers [11]. PUFs are generally classified as flexible foams or rigid foams; nevertheless, other classifications can be used, such as flexible PU slabs, flexible molded foams, reaction injection molding (RIM), carpet backing, or two-component formulations [7,11], etc. The durability and versatility of PUFs led to their use as domestic and industrial applications and even to space travel and medicine. For these reasons, the use of PUFs has grown impressively over the years [1,11].

### 2.1. Effect of the Formulation

The versatility of PUFs application arises from a wide range of properties being achievable by small modifications of the formulation used. In that sense, it is common to adjust the type and content of the polyol, isocyanate, catalyst, surfactant, blowing agent, and additives in order to control the properties of the ensuing foams. For example, the statistical evaluation of the effect of the formulation used in the production of the foams derived from crude glycerol has been reported [18]. Although the preparation of flexible foams and rigid foams involves a similar chemistry, the differences in their properties are associated with the differences of the reactants characteristics, namely those of the polyols used and of the isocyanates [7,14,19].

Several properties of PUFs can be controlled by varying the functionality of the polyol. For example, increasing the polyol functionality without changing the molecular weight leads to a slight increase in foam hardness and a small reduction in the tensile strength, tear strength, and elongation. In turn, increasing the equivalent weight of the polyol (molecular weight divided by the functionality) while maintaining the functionality of a polyol increases the tensile strength and elongation [7].

Besides the effect of the type of polyol used, the nature of the isocyanate is equally important. The most commonly used isocyanates are methylene diphenyl diisocyanate (MDI) and toluene diisocyanate (TDI). They represent about 90% of the total diisocyanate consumption [20]. Aliphatic isocyanates are also used, such as isophoronediisocyanate (IPDI) or hexamethylenediisocyanate (HMDI), but they are mainly used in other applications, such as coatings. Nonetheless, A. Sousa et al. [21] used hexamethylene-1,6-diisocyanate (HDI) to produce biofoams from castor oil and cellulose microfibers for energy absorption impact materials. PUs derived from aromatic isocyanates normally present higher glass transition, modulus, and tensile strength, but present lower elongation at break and impact resistance. In turn, PUs derived from aliphatic isocyanates are normally rubbery materials with the higher elongation at break and lower tensile strength [22].

As mentioned before, the isocyanate reacts with the polyol yielding urethane groups, and with water, yielding urea groups and CO_2_. Whilst the urethane and urea moieties are associated with the hard segments of PUFs, the polyol forms the soft segments [23]. Therefore, higher isocyanate amounts afford more hard segments, making the PUFs more rigid. However, due to health issues, MDI and TDI have been the subject of intensive research, in terms of both human and animal toxicological studies [14]. One important reason is that diisocyanates may cause asthma in sensitive individuals at extremely low concentrations [20]. MDI and TDI are included in diverse regulatory listings of dangerous chemicals because of this factor. For this reason, the banning of isocyanates is an issue that PUs producers must consider, so it is important to find alternatives in the near future, as will be discussed later.

The blowing agents are involved in the formation of the cellular structure of PUFs. There are two main types of blowing agents: (i) physical blowing agents (such as solvents with low boiling point: pentane, acetone, or hexane) which expand the polymer by vaporization; and (ii) chemical blowing agents (such as water), which expand the polymer by the CO_2_ produced [24]. The production of PUFs was greatly increased in the late 1950s by the discovery and use of chlorofluorocarbons (CFCs) as a physical blowing agent [24]. Due to the excellent chemical and thermal stability and low cost, CFCs were the blowing agent of choice for all of the PU foams, especially rigid thermal insulation foams [24]. However, later, it was proven that CFCs destroy the ozone layer [1,24]. This led to the development of an international protocol, known as the Montreal Protocol, which restrained the production and use of substances that deplete the ozone layer [11,24]. As a result of this, CO_2_ and pentane are now commonly used as a blowing agents. Pentane (*n*-pentane, *iso*-pentane, or cyclopentane) is a physical blowing agent type that is considered a zero ozone-depleting potential (ODP) blowing agent [24]. It has been used since the mid-1990s, for example, in the production of continuous rigid-faced lamination where the most stringent fire and thermal conductivity performance are required, in the production of shoe soles, or low thermal conductivity PUFs [24,25].

Although used in smaller proportions, another important reactant that is used in the production of PUFs is the surfactant. A typical surfactant is a copolymer that is composed of a silicone backbone and poly(ethylene oxide-co-propylene oxide) grafts. Surfactants lower the surface tension, emulsify incompatible formulation ingredients, promote the nucleation of bubbles, stabilize the cells, and have a significant effect on the cell size and foam air permeability [1]. They also determine the open cell content by controlling the rate of window film thinning (through drainage into the struts) and preventing rupture by localized thinning until the cell-opening event occurs.

Finally, catalysts are used to promote the reaction between isocyanates/polyols and isocyanates/blowing agents. The most common catalysts are the amines or tin catalysts [14], and a proper expansion is obtained through the balance between the polymerization (gelling) and the gas generation (blowing). In that sense, the adjustment of catalyst type and quantity dramatically influence the expansion [1,11].

### 2.2. Processing Technology

Depending on the properties or the applications needed, different technologies can be used to produce PUFs, with the most often used at large scales being the molding, the slabstock, and spraying [7].

In the molding process, the reactant mixture is injected into a mold cavity. After curing, the molded foam is removed from the mold; this is the process that is commonly used to produce seat cushions for automobiles and furniture [2]. Slabstock foams are produced by continuously pouring the reactant mixture in a moving conveyer. Cushioning and comfort materials are usually produced by slabstock methods [1]. PU spraying consists of projecting the PU in a surface or a cavity. It is normally used to produce insulation layers on flat surfaces such as roofs, but also onto nonflat surfaces such as spherical tanks and pipes and building structures [11].

Although the production of PUFs is a well-established technology, due to the need to use new raw materials and/or fillers, as well as develop products with new forms, shapes, and dimensions, new production technologies may be required in the future. In fact, additive manufacturing (such as three-dimensional (3D) printing) is already emerging as a new technology that uses different materials such as polymers, composites, metals, or ceramics and has been used to produce biomedical, aerospace, automotive, electronics, architecture, fashion, or domestic products [26]. One of the many advantages of additive manufacturing is that it can produce objects that are too complex for a traditional manufacturing process [26]. Since additive manufacturing is being widely used, the PUFs industry must pay attention to this technology to avoid becoming obsolete. In fact, Changfeng Ge et al. [27] have already reported a preliminary study of the properties of a 3D printed PUF and stated that these materials presented similar resilience properties to bulk rubber, but its density was four times lower than that of rubber. Moreover, this technology allows the possibility of printing products in customized sizes and shapes, which offers engineers an opportunity to design new cushion materials that are tailored for packaging applications.

## 3. Strategies toward the Sustainability of PUFs

### 3.1. Renewable Polyols for the Production of PUFs

Most of the polyols that are used in the production of PUFs are derived from petroleum feedstocks, but the increasing concern over the environmental impact and scarcity of petroleum in the future has motivated the development of PUFs from bio and renewable raw materials [28,29]. The growing interest for the use of renewable materials has led to an increasing use of renewable and sustainable products, such as green and bio-based polyols, in the production of PUFs. As a result of that, the green and bio-polyols market is expected to be worth 4.7 billion USD by 2021, with the North American region being the largest market for green and bio-based polyols [30]. This is due to the growing preference for the use of materials that have a lower carbon impact and are recyclable or derived from non-polluting resources. Renewable polyols are thus an obvious alternative for petroleum-based polyols. Companies such as Dow Chemical, Bayer Material Science, BASF SE, or Shell Chemicals Ltd are already commercializing bio-based polyols [31]. Moreover, extensive research has been concentrating on developing bio-based polyols from renewable sources, such as biomass residues, vegetable oils, or industrial by-products [18,32,33,34,35].

**Figure 2:**
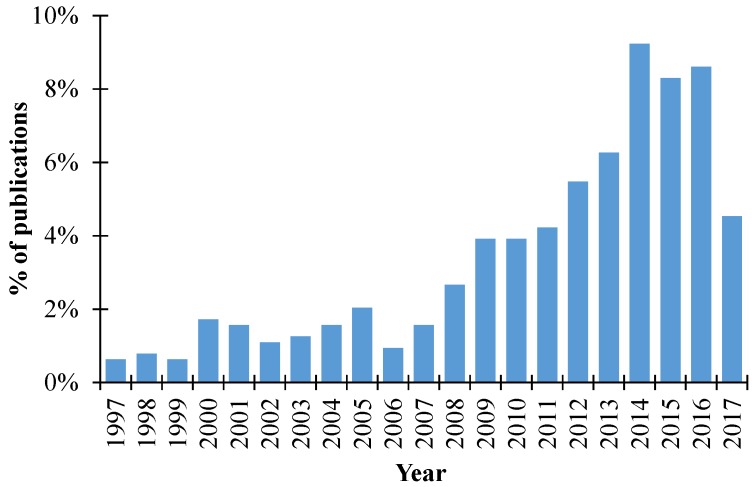
Percentage of publications searched by the Web of Science related to the use of renewable feedstocks for the production of polyurethane foams (PUFs).

The increase of research in this field is expressed by the percentage of publications (in Web of Science) that is related to the use of renewable feedstocks for the production of PUFs over the last 20 years (Figure 2).

#### 3.1.1. Use of Biomass for the Production of Polyols

Processes such as oxypropylation [36] or the acid liquefaction [37] of biomass feedstocks have been widely used to produce renewable polyols for the PU industry. The results have demonstrated that replacing, at least partially, the typical petroleum-based polyol with this type of material afforded foams with comparable foaming kinetics, density, cellular morphology, and thermal conductivity [36,37].

The oxypropylation of biomass involves an initial degradation process that is followed by polymerization that forms grafts of poly(propylene oxide) [36]. It consists in the ring-opening and anionic polymerization of oxiranes. In other words, it consists of grafting and the chain-extension of macromolecular structures containing hydroxyl groups [38]. Notice should be made that the oxypropylation does not increase the number of hydroxyl groups, but it does increase their functionality [38]. This process involves the functionalization and oxypropylation of the biomass, which can be performed simultaneously or separately [39]. In the two-step process, first, the biomass is impregnated with an alkaline solution (normally a strong Brønsted base—for example KOH in ethanol), under N_2_ atmosphere and pressure corresponding to the degradation and functionalization step. Its objective is to increase the reactivity of the hydroxyl groups that are present in the biomass [38]. In the second step, the propylene oxide is added to the functionalized biomass, yielding oxyanion groups at the end of the chains and subsequently polymerization via ring-opening anionic polymerization takes place [40]. When the propylene oxide is all consumed, the pressure drops, meaning the end of the reaction. In the single oxypropylation step, all of the reagents are added to the reactor before raising the temperature, and no solvent (such as ethanol) is used. In both cases, the final product is a mixture of oxypropylated biomass, unreacted biomass, and poly(propylene oxide). Their relative amounts in the final product are strongly related to the biomass granulometry, catalyst/biomass ratio, propylene oxide/biomass ratio, functionalization conditions, temperature, pressure, and time [41].

The oxypropylation of biomass or biomass-derived compounds such as sugar beet pulp [42,43,44], cork [45,46,47,48,49,50], chitosan [51], cornstarch [52], data seeds [53], rapeseed cake residue [54], olive stone [55] and lignin [56,57,58,59], among many others, has been carried out successfully and used to produce PUFs [36].

Ning Yan et al. [60] synthesized bio-polyols through the oxypropylation of bark, and the ensuing polyol was used in the production of PUFs. A bark pretreatment was used that consisted of activating the biomass with a solution of KOH in ethanol for 1 h at 100 °C and 50 psi. After drying, the pretreated bark and propylene oxide were added to a reactor, and the reaction was carried out for 2 h at 180 °C. The yield of this reaction was 79%, and the ensuing polyol had a hydroxyl number of 444 mg_KOH_/g. To demonstrate the potential of oxypropylated bark-based polyols in the production of rigid PUFs, the bark oxypropylated-derived foam was compared with a polypropylene glycol/glycerol-derived foam, and it was reported that the bark-derived foam presented a higher elastic modulus and compression strength. Luc Avérous et al. [61] used gambier tannin-based polyols that were obtained by the oxypropylation process in the production of PUFs. The tannin was pre-activated using a KOH solution, the oxypropylation temperature was 150 °C, and reaction was considered to be complete when the pressure returned to nearly zero. The oxypropylated tannin polyol presented a hydroxyl number of 256 mg_KOH_/g, and was used as a replacement of commercial polyols. The resulting foams were extensively characterized, and it was reported that besides having similar kinetics, the use of higher contents of oxypropylated tannin polyol afforded foams that presented a higher closed cell content and compression strength at 10% deformation, as well as lower thermal conductivity. Also, the flame resistance behavior was improved, with the addition of the oxypropylated tannin polyol.

Although oxypropylation is a suitable process to produce alternative polyols for the production of PUFs, as it requires propylene oxide, it is necessary to use homologated equipment that is capable of ensuring safety conditions against explosions provoked by sudden uncontrolled exothermic polymerizations [38]. Therefore, it is much safer to use another process of converting biomass into polyols. The acid liquefaction technique is a very attractive alternative, because it does not require the use of high pressure or hazardous reactants such as propylene oxide. The acid liquefaction of different kinds of biomass such as corn stalk [62], lignin cellulosic compounds [37,59,63,64,65,66], wheat straw [67], cork [32,68], corn [62,69,70], bamboo [71,72], date seeds [53], eucalyptus and pine woods [73], sugar-cane bagasse [74], or spent coffee grounds [75], among many others, has been successfully carried out as well.

The acid liquefaction operates under moderate temperatures (~160 °C) and at low or even under atmospheric pressure [76]. The acid liquefaction of lignocellulose materials uses liquefying solvents such as phenols or polyhydric alcohols and organic or inorganic acids as catalysts [37]. Moreover, as it will be discussed later, the liquefaction of biomass can be carried out using a biodiesel sub product (crude glycerol—CG) as a substitute of petroleum-based polyhydric polyols, which enhances the renewability of the process [65,77]. In contrast to oxypropylation, the liquefied product has a higher OH number [76]. However, the process of biomass acid liquefaction can be very complex, because smaller compounds are produced by hydrolysis, dehydration, dehydrogenation, deoxygenation, decarboxylation, rearrangements through condensation, cyclization, or polymerization, leading to new compounds [76]. Nevertheless, degradation and repolymerization are the main reactions that occur in the acid liquefaction. Despite the complexity of the reaction mechanism, the acid liquefaction is an effective method to convert biomass into polyols.

The residue content of the final product is one important issue for the acceptability of the biomass acid liquefaction, and is highly correlated with the reaction conditions such as the biomass/solvents ratio, biomass/catalyst ratio, reaction time, and temperature [37]. Degradation makes the biomass decompose and reduces the residue percentage, whereas the repolymerization of the degraded products results in insoluble materials, which increases the residue content. At the early stages of the reaction, the degradation plays a more important role, resulting in the decrease of the residue content. In turn, in the following stage, the repolymerization has a higher influence, because it is enhanced by the increase of small molecules derived from the degradation stage, which repolymerize into the insoluble polymer. So, the residue content drops at an initial stage, but it can increase after that. Therefore, the acid liquefaction time is one of the most important factors affecting the residue content.

Mona Nasar et al. [74,78] optimized the sugar-cane bagasse liquefaction process, and subsequently used this biomass-derived polyol in the production of PUFs. Mixtures of ethylene glycol and phthalic anhydride were used as solvent, and the optimal conditions for the liquefaction were 160 °C during 180 min. The effect of the partial replacement of polyethylene glycol by the sugar-cane bagasse-derived polyol in the properties of PUFs were addressed, and it was reported that the foam density and compressive strength were improved with the increasing of the biomass-based polyol content. Also, the presence of the sugar-cane bagasse-derived polyol decreased the thermal conductivity of the foams. Hong-Zhang Chen et al. [67] liquefied wheat straw for the preparation of biodegradable PUFs. Glycerol was used as the solvent, and the effect of the liquefaction reaction conditions was evaluated (influence of temperature, solvent/wheat straw ratio, catalyst content, and the water content of wheat straw). The optimal conditions were achieved using a solvent/wheat straw ratio of 6:1 and sulfuric acid concentration of 5%, at 140 °C over 2 h, and the ensuing polyol presented a hydroxyl value of 46 mg_KOH_/g. The foam prepared using the liquefied wheat straw polyol was compared with a glycol-based foam, and it was reported that the apparent density, resilience rate, elongation, and tensile strength were similar. Additionally, the water absorption of wheat straw-derived PUF was much higher, and presented good biodegradability. The degradation velocity essays have shown that the wheat straw-based foam presented a mass loss of PUF of 16%, while the glycol-derived foam had very little mass loss at the same conditions.

#### 3.1.2. Use of Vegetable Oils as Polyols

Other eco-friendly raw materials can be used as polyols in the production of PUFs. Vegetable oil-based polyols are abundant, and are usually oligomers with a wide distribution of molecular weight and a considerable degree of branching, making them a very important resource for polyols. Different kinds of vegetable oils have been used in the production of PUFs, such as castor oil [79,80,81,82,83,84,85,86,87,88,89,90], palm oil [91,92,93,94], soybean oil [28,77,95,96,97,98,99,100,101,102,103,104], rapeseed oil [105,106], canola oil [107], and tung oil [108], among others. These oils have versatile compositions and structures, are biodegradable and environmentally-friendly, are soluble in most of the industrial solvents, allowing blending with conventional petrochemical-based polyols, and the resulting foams exhibit good properties, such as flexibility, mechanical strength, abrasion resistance, toughness, adhesion, chemical, and corrosion resistance [109].

The different triglyceride structures enable the design of various raw materials for different applications. Their carboxylic acids, esters, double bonds, active methylenes, hydroxyls, oxirane rings, and others are suitable for the production of polyesters, alkyds, epoxies, polyethers, polyesteramides, or PUs [110]. The vegetable oil-derived polyols that are to be used in the production of PUs can be obtained by the functionalization of the double bond (via epoxidation, hydroformylation, or ozonolysis) or through the formation of ester bonds (such as transesterification), among others [109,111,112].

**Scheme 3:**
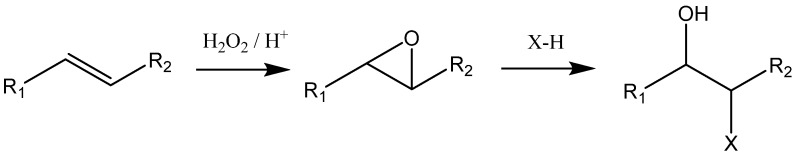
Epoxidation reaction of vegetable oil followed by oxirane ring-opening.

Epoxidation is a method that is commonly used for the functionalization of carbon–carbon double bonds. In fact, the epoxidation of soybean, rapeseed, linseed, olive, corn, safflower, karanja, melon seed, and cotton seed have been carried out on an industrial scale to produce a variety of polyols [109]. The epoxidation is usually conducted at temperatures between 30–80 °C for 10–20 h, depending on the type of feedstocks and the ratios of the reactants that are involved in the reaction. Polyols are then produced from epoxidized vegetable oils by oxirane ring-opening reactions using active hydrogen-containing compounds such as alcohols, inorganic and organic acids, amines, water, and hydrogen, as described in Scheme 3 [113].

Where *R*_1_ and *R*_2_ are fatty acid moieties of the vegetable oil (polar and apolar, respectively). A higher degree of unsaturated vegetable oils create polyols with a higher hydroxyl number, which results in higher cross-linking density and higher tensile strength PUs [113]. Yuan-Chan Tu et al. [114] produced rigid and flexible PUFs using epoxidized soybean oil as partial substitute of petroleum-based polyols. Regarding the rigid PUFs, no significant changes in density were observed, but the compressive strength decreased, while the thermal conductivity decreased first, and then increased with the increase of epoxidized soybean oil content. In turn, the density and deflection properties of flexible PUFs were similar or better than those of the control flexible foams; however, the resilience and 50% constant deflection compression properties were inferior. 

Hydroformylation is used to obtain polyols with primary hydroxyl groups, and can be applied to vegetable oils or fatty acid derivatives. In this method, the double bonds of the unsaturated vegetable oil are functionalized with carbon monoxide and hydrogen to produce aldehyde groups, which are subsequently converted to hydroxyls groups by hydrogenation (hydroformylation is usually carried out in the presence of rhodium or cobalt-based catalysts) [109,111]. The advantage of utilizing triglycerides is that high functionality and high molecular weight polyols are obtained, while in the case of fatty acids, the functionality is one-third of the triglyceride polyols, but the purification process is easier, and the obtained product presents lower viscosity [111]. PUFs derived from hydroformylated polyols can present a shorter gel time and better curing efficiency compared to epoxidized polyol PUFs [113]. Zoran S Petrovic et al. [115] produced polyols with weight average functionality varying from 5 to 2.5 that were derived from soybean oil by hydroformylation. It was reported that the heterogeneity of functionalities in polyols had no negative effect on the properties of glassy PU, but compromised the mechanical properties of the rubbery ones. It was also stated that soybean oil polyol had value and versatility, which supports the further exploration of their refinement and industrial applications.

Polyols with terminal hydroxyl groups can be obtained by ozonolysis, which involves splitting the double bods using ozone [109]. Typically, this process involves two main steps: the formation of an ozonide at the unsaturation sites of vegetable oils, and its decomposition into aldehyde and carboxylic acid. So, in this reaction, aldehyde groups are produced, which are then converted to hydroxyls in the second step (hydrogenation) by the reduction of aldehyde into alcohol using a catalyst, which is for example nickel-based [116]. The ozonolysis of soybean oils results in triols, triglyceride diols, and some mono-ols, and the polyols obtained via this route have longer network chains than the polyols prepared by epoxidation due to an extra carbon at every double bond. Zoran S. Petrovic et al. [117] also used canola and soy-based vegetable oils to produce polyols by ozonolysis. The ensuing ozonolyzed canola oil-derived PU was glassy, while the soy counterpart was a hard rubber. The temperature of the glass transition (*Tg*) of ozo-soy PU is at room temperature, and this material displayed properties that were consistent with a hard rubber such as a lower modulus, an excellent strength for a rubber, as well as high elongation. The canola polyol originated PUs with jeopardized mechanical properties. In another report, Zoran S. Petrović et al. [111] obtained polyols by ozonolysis whose molecular weight was 40% lower than that of polyols obtained by epoxidation or hydroformylation and presented low viscosity. Moreover, the ozonolysis-derived polyol resulted in clear PUs with excellent mechanical properties.

In a similar way, progress has been made in the development of polyols through ester bond reactions. In transesterification, the triglyceride is reacted with an alcohol to yield another ester group via alkoxy moiety interchange. This reaction can be catalyzed by acids, bases, or enzymes. Several aspects, including the type of catalyst, the alcohol-to-vegetable oil mole ratio, the temperature, and the free fatty acid content influence the progress of the reaction [109]. Vinícius B. Veronese et al. [118] produced polyols by the transesterification of castor oil with triethanolamine or glycerin, and the resulting polyols were used in the production of rigid PUFs. An increase of the OH number of the polyols was reported, as well as a decrease of the molecular weight and viscosity. The mechanical properties of the foams produced from the modified vegetable oil were slightly compromised in relation to those of the commercial PUFs that were used as a reference, but proved to be a potential renewable raw material for the production of rigid PUFs.

As discussed so far, an important class of vegetable oils for the production of PUFs are those that are unsaturated, such as soybean oil, sunflower oil, safflower oil, corn oil, linseed oil, olive oil, tung oil, castor oil, and others [10]. Among these, castor oil is particularly promising, as it does not compete with food supplies. In fact, castor oil, which is the triglyceride of ricinoleic acid, was of paramount importance at earlier stages of the PU industry, even before petroleum-derived polyols were used. It is extracted from the seeds of the plant Ricinus communis, and has 18 carbon atoms, a secondary hydroxyl group (C_12_), and a double bond (C_9_–C_10_). It has a functionality of around 2.7 OH groups/mol, and a hydroxyl number of around 160 mg_KOH_/g. It can be used in almost every PU application such as: coatings, cast elastomers, thermoplastic elastomers, rigid foams, semi-rigid foams, sealants, adhesives, flexible foams, and so on.

Yonghong Zhou et al. [84] reported the synthesis of castor oil-based flame retardant polyols, which involved alcoholysis using glycerol and epoxidation using formic acid and hydrogen peroxide, followed by a ring-opening reaction with diethyl phosphate. The modified castor oil was used in the production of PUFs, and the thermal degradation and fire behavior of PUFs were investigated by limiting the oxygen index, the cone calorimetry test, and thermogravimetry analysis. The foams presented regular cell sizes, and the compression strength of PUFs was improved with the increase of flame-retardant polyols. Moreover, the PUFs prepared from castor oil-based flame retardant polyols had excellent fire resistance. Maria Kurańska et al. [119] studied the influence of rapeseed oil-based polyols on the foaming process of rigid PUFs, and reported that the replacement of a petrochemical polyol by a rapeseed oil-based polyol affects the foaming process of PUFs by reducing the reactivity of the system.

The electrical and thermal conductivities of castor oil-derived PUFs were also enhanced by the addition of expandable graphite (EG) [35]. The chemical and structural characteristics of the ensuing composite foams were evaluated, as well as their morphology and their mechanical, thermal, and electrical properties. The DC (direct current) electrical conductivity results have been fitted to the Taherian model, and it was observed that EG loadings between 0.5–1.5% (*w/w*) caused a systematic increase of the thermal and electrical conductivities.

#### 3.1.3. Use of Industrial Residues as Polyols

Besides the use of bio-polyols, the use of industrial by-products, namely crude glycerol (CG), has been explored as a polyol to produce rigid PUFs. Glycerol can be obtained as a sub product of many reactions, such the saponification or the hydrolysis of triglycerides [120]. The feasibility of the conversion of CG to added value derivatives relies on several factors, such as the production cost, process technology, suitable separation and purification, and suitable CG feedstock quality [121]. In addition, the successful replacement of petrochemicals by CG in the production of PUFs has the potential to reduce their cost and environmental impact. The production of PUFs from CG might also contribute to alleviating the possible CG glut and improving the sustainability of the PUFs industry.

Originally, CG was used as a replacement for the traditional polyhydric alcohols in the acid liquefaction of biomass [65,77,122,123,124,125]. It was also used in combination with petroleum-based polyols in the production of PUFs [126]. The characteristics of the obtained polyols proved to be suitable for the production of PUFs. Yebo Li et al. produced PUFs that were derived from polyols obtained from the liquefaction of soybean straw [77] and corn stover [65] using CG as a liquefaction solvent, and reported that the obtained biopolyols and PUFs presented properties that were comparable to their analogs, which were derived from petroleum solvent-based liquefaction processes.

Being a polyol itself, CG consists of a potential raw material for the production of rigid PUFs [18,125,127,128,129]; a couple of processes regarding the treatment of CG and subsequent use in the production of PUFs have been patented [123,124]. As an alternative, CG has been used directly without any pretreatment or purification step in the production of PUFs [34]. Typically, CG contains various impurities, including fatty acids, alkoxide salts, inorganic salts, “matter organic, non-glycerol” (MONG), water, or unreacted methanol, with its composition being dependent of the feedstock used. Thus, the variable composition of CG can affect the properties of the ensuing PUFs [130]. For example, the presence of branched fatty acid and ester chains can reduce the degree of microphase separation and stabilize the bubbles during the foaming process [130]. This can result in a more homogeneous cellular structure, which can improve all of the physical properties. However, the presence of methanol is particularly important, because it can react with the isocyanate groups affecting the *R_NCO/OH_* and thus the cross-linking density; it can also be volatilized, and it may also lead to the hydrolysis of alkyl esters.

Due to the low average molecular weight of CG, the polymer formed by the reaction of CG with aromatic isocyanate has a high concentration of hard segments [130], which can increase the friability of the material.

The use of acid liquefied or oxypropylated biomass was already discussed, but other methods to use derivatives of biomass can be applied to produce PUs. One example is the use of crude tall oil. Viesturs Zeltins et al. [131] used crude deciduous tree tall oil as a polyol component to produce low water absorption rigid PUFs. The densities of the ensuing foams was in the range of 44–101 kg·m^−3^, and presented good compression characteristics and low water absorption, which have the potential to be used in boat construction or in the production of lifesaving equipment. Similarly, Kamila Mizera et al. [132] produced urea–urethane elastomers using tall oil-based polyols. It was observed that the incorporation of the tall oil polyols resulted in an improvement of the thermal and mechanical properties of the materials. Moreover, the performance as a fire retardant and evaluation of the flammability of the elastomers was also enhanced, as the heat release rate was up to three times lower than that of the reference material.

Vilas D. Athawale et al. [133] produced PU dispersions based on sardine fish oil. The sardine fish oil-derived film exhibited excellent adhesion, impact, flexibility, and chemical resistance, resulting in a cost-effective alternative to producing coatings for various applications. Even though it was used to produce PU dispersions, sardine fish oil can be a potential raw material to the production of PUFs.

Manisha S. Pawar et al. [134] used algae oil derived from chlorella microalgae to prepare bio-based polyols, and subsequently produced rigid PUFs. The bio-based polyols were prepared from algae oil via oxidation using the environmentally-friendly reagent hydrogen peroxide and acetic acid followed by epoxy ring-opening using lactic acid (LA-AOP) or ethylene glycol (EG-AOP). The epoxidation of algae oil was carried out, which led to PUFs with similar thermal properties to the foam that was prepared from commercial petroleum polyols.

Sang K. Park et al. [135] produced flexible PUFs using soy proteins that were synthetized by reacting proteins with glycerol propylene oxide polyether triol. From the results, it was observed that the density, compressive stress, resilience, and dimensional stability of foams increased with the increase of the soy protein content. 

P. K. Roy et al. [136] recycled poly(ethylene terephthalate) (PET) wastes to obtain raw materials for the production of PU–polyisocyanurate foams. This method consisted of two steps. (i) The first step involved the glycolytic depolymerization of PET in the presence of diethylene glycol (DEG) using microwave radiation in order to reduce the energy intensiveness of the process. (ii) In the second step, the glycolysate was reacted with two different diacids (adipic and sebacic acid) to obtain aromatic oligoesters. The oligoesters were then used to produce PUFs that presented cells with uniform dimensions and whose flexibility was directly proportional to the chain length of the spacer molecule that was used in their preparation. Moreover, the use of PET glycolysate in the formulation led to an improvement in the thermal stability of the resultant foams due to the introduction of phenyl rings within the polymeric chains.

A different approach was used by other authors who, instead of using industrial residues for the polyol component, used industrial residues as fillers to enhance the properties of the PUFs. Sylwia Członka et al. [137] utilized wastes that were generated by the leather industry—namely buffing dust—as reinforcement filler in rigid PUFs. It was observed that the addition of buffing dust resulted in noticeable changes in several properties, such as the foam morphology, apparent density, thermal conductivity, and mechanical strength. For example, the use of 0.1 wt % of buffing dust provided higher density (36.9 kg·m^−3^), greater compression strength (216 kPa), less water uptake (9% after 24 h), and comparable thermal conductivity (0.026 W·m^−1^·K^−1^). Others authors used cellulose and lignocellulosic fibers [138,139], egg shell wastes [140], date palm particles [141], walnut and hazelnut shells [142], or esparto wool [143], just to mention a few, as reinforcing materials in the production of PUFs, as will be discussed later.

The considerable efforts that have been devoted to the use of several industrial residues in the future can offer the possibility of using them, either directly or modified, to produce flexible or rigid PUFs with properties that are comparable to those of petroleum-based analogs.

### 3.2. Alternatives to the Use of Isocyanate

So far, it has been widely proven that fossil polyols can be easily replaced by renewable resources. However, the isocyanate still depends on petroleum feedstocks, and is a toxic reactant whose replacement and/or substitution has been far less studied than polyols. Nevertheless, studies regarding the production of isocyanates from alternatives resources such as amino acids have been carried out successfully. Also, a new type of isocyanate-free PU has been developed. Indeed, non-isocyanate polyurethanes (NIPUs) are a novel kind of PU, which present increased chemical resistance and lower permeability, as well as improved water absorption and thermal stability. Moreover, NIPUs are not sensitive to moisture in the surrounding environment [144]. NIPUs have also attracted increasing attention because of their improved porosity, water absorption, and thermal and chemical resistance over conventional PUs. Their potential technological applications include chemical-resistant coating, sealants, foams [145], etc.

One of the key reactants for the preparation of NIPUs, amines, can be obtained from commercially available products. For example, ethylenediamine, hexamethylenediamine, and tris(2-aminoethyl)amine, etc., can be utilized for NIPU preparation [146,147].

Another key reactant for the preparation of NIPUs are cyclocarbonates. The application of cyclocarbonates as reactive intermediates and inert solvents has been promoted dramatically in recent years [148]. Typically, the cyclocarbonate reactant that is used in NIPUs preparation is an oligomer or a mixture of oligomers comprising a plurality of terminal cyclocarbonate groups [144]. Cyclocarbonates have high solvency, high boiling points, are biodegradable, and have low toxicity [148]. Additionally, cyclocarbonates exhibit a wide range of reactivities with aliphatic and aromatic amines, alcohols, thiols, and carboxylic acids [148]. Dean C. Webster et al. [149] have reported the preparation of NIPUs by the formation of hydroxyurethanes based on cyclic carbonate–amine chemistry, which consists of an alternative to the hazardous isocyanate-based PUs’ chemistry.

**Scheme 4:**
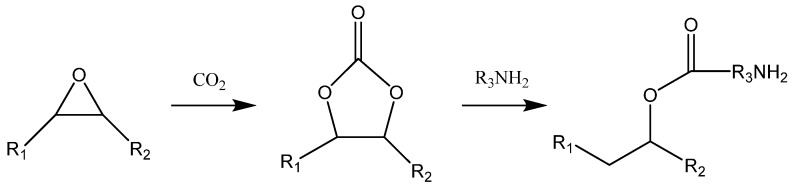
Cycloaddition of carbon dioxide into epoxidized soybean oil (ESBO) and subsequent aminolysis of cyclocarbonated soybean oil (CSBO) using a primary amine.

According to the chemical composition and original materials, NIPUs can be classified as linear NIPUs, a hybrid non-isocyanate polyurethane (HNIPU) network, renewable resources-based NIPUs, and chemically modified and nanostructured NIPUs [145]. Although linear NIPUs can find use in many applications, their mechanical properties and chemical resistance to aqueous solutions of acids and alkalis are unsatisfactory, since they lack the cross-linked network structure. The drawbacks of linear NIPUs can be minimized by the HNIPU network. HNIPUs are based on the epoxy-cyclocarbonate oligomers that contain cyclocarbonate and epoxy reactive groups, and both groups can react with amines to construct a network structure [144,150]. The formation of HNIPUs consist of multistep processes. First, there is the insertion of CO_2_ into the epoxy ring, yielding a cyclocarbonate oligomer. Then, this oligomer is reacted with a diamine, as presented in Scheme 4 [151].

Where *R*_1_ and *R*_2_ are fatty acid moieties of vegetable oils. Cyclocarbonated soybean oil (CSBO) obtained from the reaction of epoxidized soybean oil (ESBO) and CO_2_ has attracted interest for preparing NIPUs [146]. The reaction of ESBO with CO_2_ at optimized conditions resulted in CSBO in high conversion and a low level of residual epoxy [95,147]. It was reported that NIPU comprising epoxy resin, amine-curing agents, and curing accelerators could be used for coatings, adhesives, sealants, and matrices for fiber-reinforced composites with desirable mechanical and thermal properties [152,153,154,155].

Recently, James H. Clark et al. [156] produced renewable self-blowing non-isocyanate PUFs from lysine and sorbitol. The copolymerization of a sorbitol-derived bis-carbonate with diamines from lysine under solvent-free conditions was used to produce rigid PUFs. It was stated that the CO_2_ produced remains trapped within the bubbles rather than being released into the atmosphere, meaning an improvement of the sustainability of foams. Moreover, the materials produced proved to be almost 100% bio-based, inexpensive, and of low-toxicity, with potential applications for insulation and packaging.

Abdolreza Farhadian et al. [157] synthesized non-isocyanate poly (ester amide/urethane) networks that were derived entirely from vegetable oil without using solvent, which had no rigid nor aromatic structures to improve their thermal stability. These NIPU networks showed good thermal stability, low water absorption, and degradation. Thus, the results presented the potential of this environmentally-friendly strategy for preparing bio-based NIPU for high performances. Furthermore, the presence of aliphatic ester groups and their biodegradable nature may also make them proper for biological and/or biomedical applications. These non-isocyanate poly (ester amide/urethane) networks can have potential application in the production of PUFs.

Similar to how the CFCs blowing agents were banned by the Montreal Protocol, as well as the dioctylphthalate (DOP) that were used as plasticizers in the PVC industry, the banning of isocyanates is a possibility that can become a reality in the future and for which the PU industry must be prepared. The challenge is then to use the NIPUs at a large scale and apply them to common PU products or even produce new NIPU materials.

### 3.3. Recycling of Polyurethane Foams

Due to the variety of their applications, the production of PUs has increased in the past decades, leading to an increase of PU wastes [158]. PUFs´ wastes generated in production or recovered after use can be disposed in landfills or incinerated, but neither are acceptable because long-range ecological goals dictate zero pollution as well as the conservation of raw materials [159]. In fact, recycling is not only a requirement for preventing pollution and environment protection, it is also needed in order to reduce production costs and increase material utilization efficiency.

There are two major methods for recycling PUs: physical recycling and chemical recycling. In physical recycling, the PU scraps are directly reused without chemical treatment, while the chemical recycling follows the degradation principle. In this case, the PU wastes are gradually depolymerized into oligomers and even smaller molecules, which can later be used as reactants to produce new PU materials [159].

#### 3.3.1. Chemical Recycling of Polyurethanes Foams

**Figure 3:**
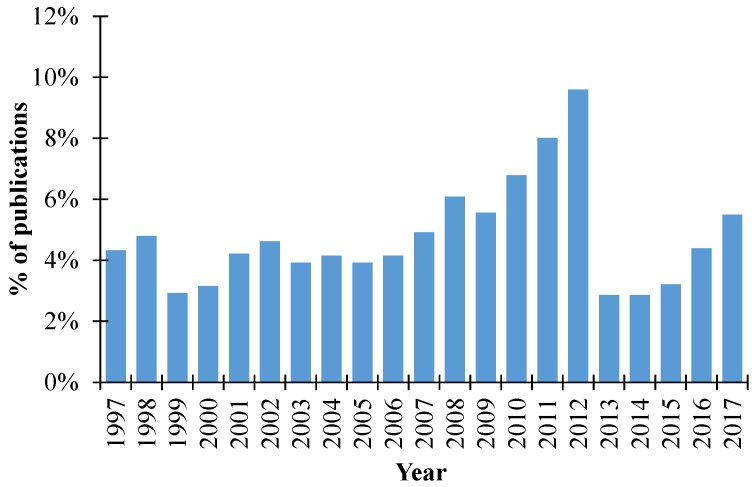
Percentage of publications searched by the Web of Science that were related to the recycling of PUs.

Many thermo-chemical methods for recycling PUs have been developed, such as hydrolysis, glycolysis, alcoholysis, and aminolysis, among others [160]. In Figure 3, the percentage of publications (in the Web of Science) related to the use of recycling of PUs in the last 20 years is presented.

**Scheme 5:**
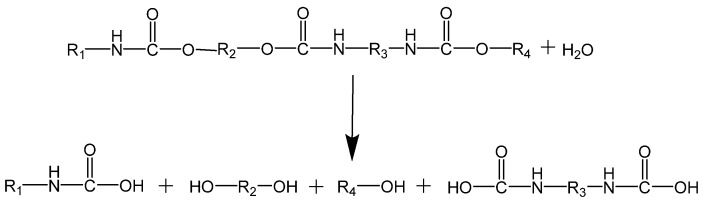
Reaction scheme of the hydrolysis of PU.

In the hydrolysis of PUs, an overheated steam is used to hydrolyze urethane bonds yielding polyols and amines [160,161], which upon separation and purification can be reused as raw materials for the production of PUs (see Scheme 5).

Suguru Motokucho et al. [162] recycled two types of aliphatic PUs (namely H-PU and I-PU which were synthesized from 1,4-butanediol and 1,6-hexamethylene diisocyanate or isophorone diisocyanate, respectively) by hydrolysis under pressured CO_2_ in a water system. The hydrolysis of PUs was dependent on the temperature and CO_2_ pressure. It was reported that 98% of H-PU and 91% of I-PU were successfully hydrolyzed at 190 °C for 24 h under 8.0 MPa. Afterwards, the final products were isolated by evaporation of the water-soluble components. In turn, Noboru Yamamoto et al. [163] applied enzymatic degradation to PUs and segmented PU ureas using various proteases. It was reported that proteases, such as papain, bromelain, and ficin showed high activity and were effective to cleave urethane bonds.

**Scheme 6:**
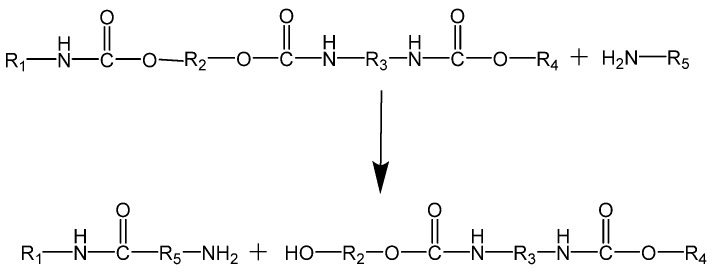
Reaction scheme of the aminolysis of PU.

The aminolysis of PUs is based on breaking urethane bonds using amines (e.g., dibutylamine or ethanolamine) affording polyols and disubstituted ureas (see Scheme 6) [164]. The final products are oligomeric ureas and amines [165].

Saowaroj Chuayjuljit et al. [166] depolymerized scraps of rigid PUFs by aminolysis using diethylenetriamine as the degrading agent and sodium hydroxide as both reactant and catalyst. The reaction was carried out at 180 °C for 70 min and yielded 4,4′-methylenedianiline (MDA), low molecular weight urethane oligomers, and other chemicals. According to the authors, these products can be subsequently separated by extraction with cyclohexane and distilled water. It was also reported that the depolymerization increased as the amount of NaOH increased. The reaction yielded MDA.

**Scheme 7:**
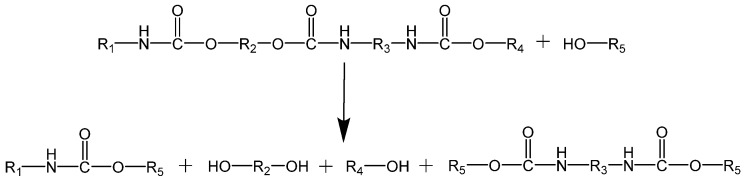
Reaction scheme of the alcoholysis of PU.

Alcoholysis involves the substitution of one hydrogen atom of water by an aliphatic group that affords an alcohol (see Scheme 7) [160]. The reaction is performed in a similar way as the hydrolysis at high temperature and under pressure. This process was developed aiming at facilitating the separation of the amines from the polyol/methanol mixture, but the separation is nearly as difficult as in the case of hydrolysis, and additionally, methanol has to be evaporated. Numerous patents for PU alcoholysis are available, but none of the processes have been adopted at the industrial scale [167].

Changyu Li et al. [168] recycled PUFs by alcoholysis, and it was reported that the obtained products consist mainly of glycol, propanol, and 1,2-Benzenedicarboxylic acid, which can be used as raw material for the production of PUFs. It was also reported that the higher content of glycol leads to a reduction of the viscosity of the recovered product. Moreover, new PUFs were prepared from the recovered product, but their thermal stability and mechanical properties were lower than that of the original PUFs.

**Scheme 8:**
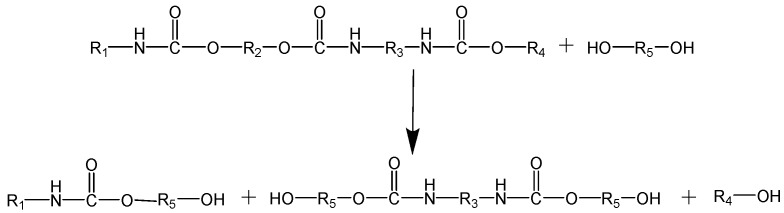
Reaction scheme of the glycolysis of PU.

The most common method, which is currently used in the industry, is the glycolysis, which uses high boiling glycols as decomposition reagents (see Scheme 8) [160,169]. Common drawbacks of PU glycolysis processes include their high-energy demands and long reaction times, which significantly restrict their utilization in industry [170].

Chao-Hsiung Wu et al. [171] recycled flexible PUs from car wastes by glycolysis. The glycolysis was carried out under atmospheric pressure and isothermal condition (220 °C) and diethylene glycol and potassium acetate were used as the solvent and catalyst, respectively. The results indicated that adequate concentrations of diethylene glycol (DEG) and potassium acetate (KAc) are about 150% and 1% of the mass of the PU, respectively, and an adequate reaction time is 90 min, but purification of the glycolysis products was necessary. Joanna Paciorek-Sadowska et al. [172] recycled rigid PUFs and rigid PU–polyisocyanurate foams by glycolysis. Afterwards, foams containing the glycolysis product were produced, which presented better flammability properties. D. Simón et al. [173] used glycolysis to recycle the wastes of viscoelastic flexible PUFs, and two phases were obtained: (i) an upper phase that was mainly formed by the recovered polyol which after purification, presented excellent characteristics to produce flexible PUFs; (ii) and a bottom phase, which was used up to 75% as a substitute of conventional polyol in the production of rigid PUFs.

**Scheme 9:**
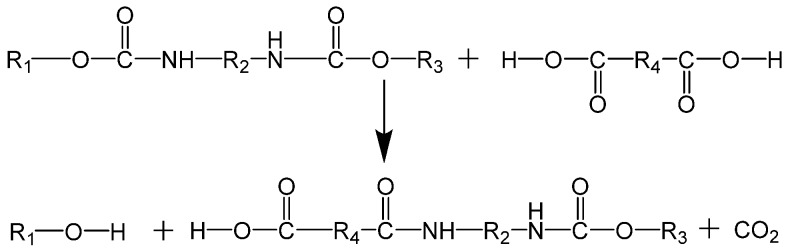
Reaction scheme of the acidolysis of PU.

All of these methods have advantages and disadvantages, but glycolysis is the most commonly used at the industrial scale. However, the separation and purification procedures are still too costly, which compromises the economic viability of this process. Moreover, a new route for recycling PU scraps is emerging—the acidolysis—which converts PU wastes into a liquid, using a cleavage agent (generally dicarboxylic acids). The cleavage agent reacts with the carbamic group bond of the PU network, which will gradually depolymerize into small molecules, which are carbon dioxide and water the reaction sub products (see Scheme 9).

Many dicarboxylic acids can be used in the acidolysis of PUs scraps, such as oxalic acid, malonic acid, succinic acid, or adipic acid. Also, they can be used singly or as a mixture of at least two, and the type and quantity of each can be adjusted/optimized to the characteristics that are required for the recovered polyol. The temperature and reaction time are important parameters, because they must be maintained within a specific range so that the obtained polyol presents suitable characteristics to be used in the production of PUFs. Low temperatures and short reaction times yield a final product with higher acid value, lower hydroxyl value, higher molecular weight, and higher viscosity. Higher temperatures and long reaction times result in a polyol with a lower acid value, higher hydroxyl value, lower molecular weight, and lower viscosity, but can darken the color of the polyol.

#### 3.3.2. Physical Recycling of Polyurethane Foams

Physical recycling only involves the mechanical action of PU scraps, and the ensuing material is reused without chemical treatment. In turn, here are several processes that use mechanical action for the PU recycling, namely rebinding, regrinding, adhesive pressing, compression molding, and injection molding.

In rebinding, PU scraps are milled and held using a binder. In fact, the rebind process has been used for a long time; 50,000 tons of rebinding foam is processed each year in Western Europe, and new applications are constantly being developed [174]. Mir Nikje et al. [174] reported that PUF scraps can be reprocessed by mixing scrap particles (size approximately 1 cm) with diisocyanate MDI, followed by form-shaping at 100–200 °C and 30–200 bar pressure, and the final product can present high density and excellent resilience properties, making it suitable for flooring, sport mats, cushioning, packaging, carpet underlays, vibration, and sound-dampening applications.

The regrind technology, which is sometimes also described as powdering, is a process where PU scraps are grinded into a fine powder and mixed with the polyol component to make new PUs. There are a number of ways to produce small particles, among which the two-roll mill process and pellet milling are the most common. The two-roll mill process consists of two rollers, rotating in opposite directions and at different speeds to create shear forces in the very narrow gap between them. The pellet mill consists of two or more metal rollers, which press the PU foam through a metal die. Cornelia Diessel et al. [175] patented the process for recycling thermosetting PUFs in which they are comminuted in a mill, and pressed under the influence of pressure and elevated temperatures to produce high-strength sheets. In turn, PU powder can be used as filler in the production of PUFs, through being added to the polyol. To be used as filler, the particle size of the PU powder should be between 100–200 μm, and different milling and knife-cutting processes are needed [176].

Adhesive pressing is probably the oldest method of flexible PUFs recycling. It consists of coating PU particles with an adhesive followed by hot pressing [174]. Wolfgang Hippmann et al. [177] developed a method in 1977 of producing low-density slabs made by blending chipped PUFs with a binder resin and molding or extruding the mixture to shape. The binder resin may also be a PU or thermoplastic resin in the form of a liquid or powder. If properly blended, a highly regular composite structure is obtained, with potential application such as sound-absorbent paving slabs. Nonetheless, the PUF scrap particles can me mixed with MDI, followed by forming at 100–200 °C and 30–200 bar pressure. The product of this method has been useful as insulation panels, carpets, and furniture.

In compression molding, PU particles are submitted to high temperature and pressure (for example 180 °C and 350 bar) in order to flow the particles together, without any binder, with the resulting compression molded parts being produced from 100% recycled material. Compression molding is capable of producing high-performance recycled products [174]. Molded PU products can contain ≤20% regrind material without the serious deterioration of physical properties, mechanical properties, or performance [176]. Nonetheless, the reprocessing of PU wastes is quite difficult, because they are not thermoplastic materials. Maciej Heneczkowski et al. [178] reported a method of reactive extrusion that is considerably cheaper than the known techniques of PU reutilization. It yields a thermoplastic granulate that can be injection molded or extruded, yielding various products. The good properties of the resulting material make it suitable for various applications such as shoe soles, gaskets and hoses (oil-proof ones), sheets, or insulating films, etc.

Although the physical recycling of PUFs has been used for very long time, its potential has not been explored extensively. The use of PU powder in PUF formulations or the production of PU composites can be an opportunity that is both environmental and economical that increases the eco-efficiency of the PUs.

## 4. Polyurethane Foams Applications and Enhancement of Properties

Despite the excellent properties of PUFs, it is common to prepare PUF composites in order to improve their properties. Besides thermal insulation, reaction to fire, and sound absorption properties, which will be discussed later, other properties such as the mechanical properties, the susceptibility to fungi in wet environments, and the electrical conductivity can be improved by the addition of functional fillers. The use of these fillers increases the range of the applications of conventional PUFs beyond the building, construction, and automotive industries to radar absorbing and electromagnetic interferences (EMI) shielding, oil absorbents, sensors, fire proofing, shape memory, or biomedical materials, especially when nanofillers are considered [179].

### 4.1. Mechanical Properties

Even though the mechanical properties of PUFs are suitable for almost every application, they can be tailored to meet the new requirements of advanced applications. Different types of fillers and nanofillers, such as cellulose and lignocellulosic fibers [138,139], glass wool, glass microspheres or glass fibers [180,181,182], egg shell wastes [140], date palm particles [141], walnut and hazelnut shells [142], and esparto wool [143], just to mention a few, can be used to improve the structural and mechanical properties of PUFs. Despite having a significant effect on the thermal and electrical properties, materials such as carbon nanotubes (CNTs) [183,184,185,186], graphene [187,188,189,190], EG [35], carbon nanofibers (CNFs) [191], or inorganic fillers, such as Fe_3_O_4_ [192], TiO_2_ [193], and iron oxide [194] are also commonly used to improve the structural and mechanical properties of the foams.

Attention must paid to the possibility of the addition of fillers to the PU matrix affecting the reaction between the polyol and the isocyanate due to at least two possibilities: (i) the hydroxyl groups on the surface of the filler can alter the isocyanate index (*R_NCO/OH_*), thus affecting the consumption of NCO groups; and (ii) the interference of the filler on the rate of the polymerization, which is namely associated with changes in the rheological behavior of the reaction mixture and/or the coupling of filler surface groups with either the isocyanate, the polyol, and/or the water if it is used as the blowing agent [195]. Therefore, the presence of fillers may lead to an increase or decrease of the urethane/urea linkages ratio, altering the cross-linking density and affecting the morphology of the foams, as well as the thermal and mechanical properties of the ensuing composites [196,197,198,199,200,201]. Also associated with the performance of composites is the effect that fillers may have on the phase separation resulting from thermodynamic incompatibility and/or interference of the fillers with the hydrogen bonding network that is commonly established between the urethane or urea groups and carbonyls in PUs [35]. Moreover, the addition of nanofillers to PUFs is also known to have variable effects depending on the percentage of loading, on the size of particles, and because fillers can be incorporated in the cell walls [5,10]. 

PUFs composites with enhanced properties can reduce the weight, energy, and cost of the material/application. In that sense, carbon-based nanoparticles such as EG, CNTs, graphene, and carbon black have attracted increasing interest due to their inherently high mechanical and physical properties [179]. The incorporation of these particles in PUFs has been known to enhance their mechanical performance [35,127,187,202]. Nowadays, due to their lower price, CNTs or graphene have been used in many applications. Following this trend, it is expected that in the near future, these carbon nanofillers will be used in the PU industry at a larger scale.

Yet, the impact of the use of fillers on recycling, health, and the environment still needs to be fully evaluated. Furthermore, the mechanical performance of these composites is dependent on the percentage of loading, the size of particles and whether the fillers are incorporated in the cell walls or not. A recent report [35] on the study of the physical properties of bio-based PUF/EG composites showed that the Young’s modulus (*E*), toughness, and compressive stress (*σ_10%_*) of the composites are not linearly related to the increase of EG. In fact, an attempt to apply the rule of mixture, for example to *E*, afforded totally uncorrelated results. Although there was an initial increase of the mechanical properties for lower loading beyond 0.75% of EG, there was a decrease in performance, which may be related to the effect that EG has on the reduction of the cross-linking density of the polymer matrix. The deterioration of the mechanical properties can be attributed to the effect of EG in the foaming process, which affects the foam microstructure, the density, and consequently, the compressive mechanical properties. In that sense, the good dispersion of these particles to achieve enough wettability of the filler’s surface and thus influence the orientation of the fillers in the matrix is of paramount importance.

### 4.2. Thermal Regulation

Normally, open cells foams are appropriate for sound insulation applications, while closed cells foams are appropriate for thermal insulation applications. The thermal insulation is primarily due to a combination of cell size and cell morphology, which trap the low thermal conductive gas inside. Within this scenario, closed cell foams, which are associated with rigid foams, are an important class of materials due to their outstanding thermal insulation properties. In addition, their high mechanical strength and their easy processing make rigid PUFs an attractive choice in various industrial applications, such as refrigerators and sandwich panels, where rigid PUFs dominate the market [1].

As mentioned before, the main application of rigid PUFs is in the field of thermal insulation. Since PUFs are composed by a small portion of polymer whose thermal conductivity coefficient (*λ* or *k*) is in the range of 0.1–0.3 W·m^−1^·K^−1^ [203], and a high portion of gas with lower thermal conductivity (*λ* ~ 0.0146 W·m^−1^·K^−1^ [204]), which is essentially trapped in the closed cell pores, the thermal conductivity of PUFs is much lower than that of the solid made of the same material. In fact, thermal insulation is associated with the reduction of the heat flux that passes through the material, which can be defined by Equation (1):

(1) $$\dot{Q}=k/d\;\left(T_{1}-T_{2}\right)\;$$

where $\dot{Q}$ is the heat flux in W·m^−2^ that passes from one face of the material to the other per m^2^ area, *k* is the thermal conductivity coefficient of the material in W·m^−1^·K^−1^, *d* is the thickness of the insulation material in meters, and *T*_1_ and *T*_2_ are the temperatures on each surface of the insulation material. *k/d* or *h* is also called the heat transfer coefficient for the insulation layer, and is expressed in W·m^−1^·K^−1^ [205].

**Figure 4:**
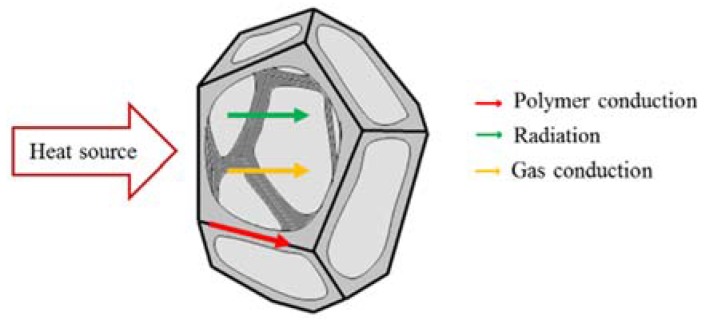
Representation of heat transfer mechanism in polymeric foams (adapted from [207]).

The thermal insulation performance of a typical closed cell PUF filled with low thermal conductive gas derives from three heat transfer mechanisms: convection, conduction, and radiation. In this kind of PUF, the heat transfer through the gas by conduction comprises 40% to 50% of the total heat transfer, whilst the heat transfer through the polymer by conduction and radiation comprises about 25% to 30% of each one [206]. So, the overall thermal conductivity coefficient results from the sum of these contributions, as expressed by Equation (2) [1]:

(2) $$\;k=k_{conduction}+k_{convection}+k_{radiation}\;$$

where *k_conduction_* is the thermal conductivity by conduction, *k_convention_* is the thermal conductivity by convection, and *k_radiation_* is the thermal conductivity by radiation (see Figure 4).

As regards heat transfer by convention, in PUFs with an average cell size below 0.5 mm, it can be considered negligible [205,208].

The thermal diffusivity of thermal insulation materials is also important for their engineering. The thermal diffusivity *D* (m^2^·s^−1^) is defined as the rate of heat that is transferred from the hot side to the cold side of a material. *D* describes how quickly a material reacts to a change in temperature; it is related to the thermal conductivity and the density of the material, and is calculated by dividing the thermal conductivity by the density and specific heat capacity according to Equation (3) [209]:

(3)  D=k/ρc 

where *k* is the thermal conductivity (W·m^−1^·K^−1^), *ρ* is the density (kg·m^−3^), and *c* is the specific heat capacity (J·kg^−1^·K^−1^) of the material.

Normally, rigid PUFs are the first-choice materials to be used for the thermal insulation of buildings and refrigerators, among others. This is because rigid PUFs presents excellent thermal insulation properties in the temperature range from −196 °C up to 130 °C [210]. In fact, rigid PUFs present lower thermal conductivity (0.02 W·m^−1^·K^−1^ or even lower) than the other thermal insulation materials that are commonly used, such as mineral wool (0.037–0.055 W·m^−1^·K^−1^), expanded polystyrene (0.03–0.04 W·m^−1^·K^−1^), extruded polystyrene (0.034–0.044 W·m^−1^·K^−1^), cellulose (0.04–0.065 W·m^−1^·K^−1^), and cork (0.04–0.05 W·m^−1^·K^−1^) [211].

Camila S. Carriço et al. [212] synthesized rigid PUFs from a mixture of castor oil and CG, which presented a thermal conductivity between 0.0128–0.0207 W·m^−1^·K^−1^. Sung Woong Choi et al. [213] analyzed the thermal properties and heat transfer mechanisms of PUFs blown with water and reported a thermal conductivity between 0.023–0.027 W·m^−1^·K^−1^. M. Kirpluks et al. [214] used natural oil-based polyols as feedstock for the production of thermal insulation rigid PUFs, which presented a thermal conductivity below 0.022 W·m^−1^·K^−1^, just to mention some recent reports that have highlighted the suitability of the use of PUFs as thermal insulation materials.

Nonetheless, the thermal insulation properties of PUFs can be improved by a better control of the foaming process. Beyond that, if heat storage materials are incorporated into PUFs, the heat gain/loss from/to the surroundings will be reduced, and the energy savings will be much more efficient [215,216,217,218,219,220,221]. In the last years, this concept has found growing interest as a result of the rise of a new class of materials: the phase change materials (PCMs). PCMs, which are also called latent heat storage materials, have the capability of storing and releasing a significant amount of thermal energy within a small temperature change or even no change. In that sense, PCMs are often used as thermal regulation fillers [222].

According to their phase change states, PCMs can be classified as liquid–gas PCMs, solid–solid PCMs, and solid–liquid PCMs. Liquid–gas PCMs have the highest energy density, but their high volume variation is a major drawback [223,224]. For that reason, the materials that are used for thermal energy storage are typically salt hydrates or paraffins.

The advantages of solid–solid PCMs, such as molecular crystals, are that a liquid phase does not need be contained, so the segregation of components does not occur [225]. Nonetheless, the most commonly used PCMs are the solid–liquid phase transition, such as paraffin waxes, poly(ethylene glycol)s, or fatty acids and their derivatives. Paraffin waxes have been used as heat storage materials, which the results have shown absorb, store, and release a great amount of heat and present high latent heat storage capacities. Moreover, they are chemically inert, noncorrosive, odorless, long-lasting, inexpensive, easily available, ecologically harmless, and nontoxic, which makes them preferred for thermal storage applications [226,227,228,229].

In solid–liquid PCMs, the energy is absorbed by the breakdown of the bonds that are responsible for the solid structure. When the materials cool down, the latent heat that was previously absorbed is released to the surroundings, and the PCMs return to their solid state [216,222,224,230,231,232]. Outside the temperature range of the phase change process, the PCM behaves as a common material, storing sensible heat [23].

Nonetheless, in general, the low thermal conductivity of paraffin-based PCMs requires the use of large and expensive heat transfer surfaces, which is considered a major drawback [233]. Additionally, in view of the price of PCMs, it would not be viable to consider loading PUFs with high amounts PCMs at a large scale. Furthermore, their low thermal conductivity, combined with that of PUFs, tends to further limit their application as thermal regulation materials. In that sense, the thermal conductivity of PUFs must be increased, and one option to achieve this is filling the polymer with conductive agents, such electron-conductive fillers, to form a network in the matrix of the polymer. Carbon-based materials such as EG present several advantages over usual metal fillers due to their lower costs, corrosion resistance, fire-retardant characteristics, and ease of processing [234]. Moreover, the addition of EG enhances the mechanical and the flame-retardant properties of the ensuing composites, which in the case of PUFs containing paraffin-derived PCMs can be an issue [127,233,235]. In turn, the lack of compatibility of EG and PCMs with the foams can disrupt the cellular structure, affecting all of the properties of the foams and ultimately compromising its applications.

### 4.3. Reaction to Fire

During combustion, PUFs generate highly toxic smoke, especially carbon monoxide (CO) and hydrogen cyanide. The inhalation of these gases causes severe health problems or even death. Also, during a fire, there is a dramatic increase of temperature, which leads to the decomposition of PUFs releasing small molecules in the gaseous phase. The mixture of these small molecules with air forms a flammable mixture, and when the concentration of this mixture and temperature cross the flammability limit, the material starts to burn [236].

The behavior of a material in connection with fire can be classified as: (i) resistance to fire and (ii) reaction to fire. The fire resistance provides information about how well a building element, such as a wall, floor, door, etc., can maintain its properties when exposed to a fire, while reaction to fire is the measurement of how a material or system will contribute to the development and spread of the fire.

There are several parameters that can be used to evaluate the reaction to fire of the materials. One of the most important is the heat release rate (HRR), which represents the quantity of heat generated per unit area and time [237]. When a sample is ignited, heat is generated, so the HRR is an index to the extent of fire [238]. The effective heat of combustion (EHC) is the ratio of the amount of heat released to the rate of mass loss measured at a certain point, and is related to the volatile gases that are formed during combustion [239]. As it is well known, compounds resulting from incomplete combustion (carbon monoxide—CO) are more toxic than those resulting from a complete one (carbon dioxide—CO_2_); therefore, the CO/CO_2_ weight ratio is an index of smoke toxicity. Finally, another parameter of great importance that needs to be considered is the specific extinction area (SEA), which is related to the smoke density. The lower the smoke density is, the easier it would be for people to escape from a fire; hence, low SEA values are desired [240].

In view of the increasing awareness of public opinion, the reaction to fire of PUFs needs to be improved, and this can be achieved by the incorporation of flame retardants [236]. Flame retardants can be used as additives or as reactive in order to interfere with combustion during different stages such as heating, decomposition, ignition, or flame spread; halogenated paraffins and phosphorus-containing compounds are commonly used flame retardants. However, paraffinic compounds may not be very compatible with the PUFs, and for that reason, they may jeopardize the mechanical properties of the materials besides releasing irritant acids. In turn, phosphorus compounds, as a reactive type flame retardant, can react with the functional groups of PUFs. They act as char-forming agents, reducing the generation of flammable gases [236]. Reactive-type flame retardants have the advantages of (i) increasing the compatibility between polymers, (ii) not degrading the mechanical properties of the PU, (iii) possessing better compatibility, as the flame-retardant group is a part of the binder, and (iv) using small amounts or low concentration for the enhancement of fire-retardancy [236].

To overcome the poor compatibility and easy migration of additive flame retardants, Wen-Hui Rao et al. [241] synthesized a novel polyester polyol from dimethyl methylphosphonate and diethanol amine through transesterification to be used as a flame retardant in flexible PUFs. It was observed that 10 PHP of the polyester polyol that was synthetized originated flexible PUFs with elevated tensile strength and elongation at break. Moreover, it was reported that the fire resistance of the materials was improved significantly with the incorporation of the polyester polyol that was synthetized.

Wei Xu et al. [242] produced rigid PUFs using a combination of nanostructured additives (zinc oxide (ZnO), Zeolite, and montmorillonite (MMT)) and phosphorus flame retardants (ammonium phosphate (APP) and dimethyl methylphosphonate (DMMP)). It was stated that the heat release rate (*HRR*) of PUFs filled with Zeolite/DMMP/APP was only 91 kW·m^−2^, which was 56% lower than that of the neat foam, and 26% lower than that of the foam filled with DMMP/APP.

Renata Lubczak et al. [243] produced boron-containing PUFs using oligoetherol as a reactive retardant, and the results showed a high oxygen index (24.7%) and high thermal resistance. Moreover, these materials showed good mechanical resistance before and after annealing at 175 °C.

Khalifah A. Salmeia et al. [244] reviewed the recent advances in 9,10-dihydro-9-oxa-10-phosphaphenanthrene-10-oxide (DOPO) chemistry, classifying its derivatives based on the chemical reactions and functional groups. The reaction mechanisms of each specific reaction for DOPO derivatization are discussed in detail in that work, as well as some characteristic applications of those DOPO derivatives. Although DOPO has been used since 1972, the recent advances have attracted a lot of attention for this type of flame retardant as a result of the need for developing halogen-free flame retardants. Furthermore, that the methods to produce DOPO are economical has definitely helped its recent commercial exploitation.

A very distinct type of flame retardants is inorganic fillers. These materials produce a stable organic–inorganic interface, which reduces the concentration of decomposition gases and increases the diffusion path barrier of the volatiles that are produced during the degradation process [236]. Nowadays, EG is widely used as a flame retardant in PUFs. Once exposed to heat, EG forms a low density “worm”-like structure on the surface of the PUFs that prevents heat and oxygen transfer, providing a good fire resistance performance [245]. The char layer, being a thermal barrier and thwarting oxygen diffusion, prevents the further decomposition of the materials. Therefore, due to this protection effect, the presence of EG can also decrease the mass loss rate and subsequently increase the residual mass of the PUFs after burning. Moreover, the expansion of this filler suffocates the flame, and the compact char layer that is formed limits the heat and mass transfer from the polymer to the heat source. According to M. Modesti et al. [246], this expansion is due to a redox process between H_2_SO_4_ (present within the graphite layers) and the inner graphite surface that generates gases according to the reaction:

$$\;C+2H_{2}{\mathrm{SO}}_{4}\to {\mathrm{CO}}_{2}+2H_{2}O+2{\mathrm{SO}}_{2}\;$$

In fact, the gases that are released can increase the volume of the materials by about 100 times. A recent report [127] where thermographic images and temperature/time plots were used has shown that the combustion of a neat PUF proceeded in a steady way, from bottom to top, and the temperature of the burning front (regions above 500 °C) was typically in the range of 600 °C, with rare regions attaining a maximum temperature of about 750 °C. The temperature of the sample decreased steadily over more than 10 s, which was the time of the complete combustion of the PU. In turn, the combustion of the PUF filled with 5% of EG proceeded in a much more inhomogeneous way. A maximum temperature of about 1000 °C was quickly attained and concentrated in the “worm”-like structure that was developed at the surface of the sample. Then, the combustion was seen to stop very suddenly at about 4.0 s, and within about 0.5 s, no point was observed that had a temperature that exceeded 450 °C. Therefore, it was suggested that EG is not only a fire retardant; it could also be seen as a flame extinguisher.

A. Lorenzetti et al. [235] reported that due to the effect of expansion volume and intercalants on the flame retardancy of EG in PUFs, the *HRR* decreased because of the heat shield formed by the expansion of the EG due to: (i) the gases that are released from the redox reaction (explained below) of EG dilute the flammable gases, suffocating the flame; and (ii) the decomposition, being an endothermic process, absorbs the heat released during combustion.

Due to its chemical and morphological characteristics, the EG can decrease the EHC value, preventing the combustion and release of heat, when the matrix is decomposed into the gas products. Jarosinski et al. have attributed this to some products released by the flame retardant possibly quenching the radicals that are derived from the matrix and preventing the combustion of original ignitable components [247].

The physical barrier effect formed by EG thwarts the oxygen diffusion, favoring the development of products of incomplete combustion, similar to CO [248]. Consequently, the higher this ratio, the higher the dangerousness of the material when burned.

Once again, the presence of EG can contribute to the decrease of the values of this parameter, meaning that it has an important role in the condensed phase.

PUFs as well as most organic materials burn very easily. Despite that, for many years, the fire performance of PUFs was suitable. However, in the future, materials will have to meet ever more stringent requirements due to the greater attention paid to fire safety; thus, improved fire performance is required.

### 4.4. Sound Absorption Properties

The extensive use of PUFs as construction materials is not just associated with their thermal insulation properties, but also with their sound-absorbing properties, especially for industrial applications, theatres, offices, sound studios, and many other applications [205].

The sound absorption coefficient (*α*) defines the energy that is absorbed when a sound wave hits a material and is determined by the ratio of the sound absorbed (*W_abs_*) to the sound incident (*W_inc_*) [249,250]. Moreover, the efficiency of a sound absorber results from the balance between a small amount of sound reflection and a large amount of sound dissipation.

Flexible foams are known to have good sound absorption properties due to the cavities, channels, or interstices that are present in their structure [205]. When sound waves flow through the open pore cells of a flexible foam, the frictional forces between the air flow and cell wall convert the sound energy into heat energy [251,252,253]. However, this does not fully explain the sound absorption capability of rigid PUFs. Indeed, there are others mechanisms to describe the sound absorption of materials: discrete, porous, and resonant (which include membranes and Helmholtz resonators, which are also known as a perforated absorber).

Discrete absorbers can literally be anything: panel, wall, person, bookshelf [253], etc. Porous absorbers are the most common, and include natural fibers (e.g., cotton), mineral fibers (e.g., glass fiber), foams, carpets, and so on [254]. When the sound waves strike the surface of a porous absorber (such as an open cell foam), the air flows through the material, forcing the cell walls to stretch, bend, and buckle. These frictional forces convert the sound energy into heat, as previously mentioned [253]. Membrane-type resonant absorbers utilize the resonant properties of a flexible membrane to absorb sound. An example of membrane absorbers is the wood panels that are mounted in front of the rigid back where the air columns inside vibrate, responding to incident sound waves. The friction between the air and the walls results in the absorption of sound energy [254]. In turn, Helmholtz absorbers consist of enclosed volumes with a relatively small aperture.

For instance, an open cell foam can act as a porous absorber, a closed cell foam can act as a membrane absorber, and an open/closed cell foam can act as a Helmholtz/perforated membrane absorber. Due to the different sound-absorbing mechanisms, the application of rigid foams as sound absorbers requires adequate understanding of the relationship between the mechanical properties, the porous structure, and the sound absorption performance [250,252,255,256]. There have been many studies that have attempted to predict it, including studies about airflow resistivity, porosity, elastic constants, pore geometry, and so on [251,253,257]. Ling et al. have described a method of production of rigid PUFs that possess high sound absorption properties, but the relationship between their acoustic properties with the stiffness of the foams was not discussed [258]. Tiuc et al. improved the sound absorption properties of rigid PU foam with the incorporation of textile wastes [259]; however, the homogeneity of the material was difficult to achieve, and the influence of their mechanical properties on the cellular structure on the sound absorption coefficient values was not discussed. Furthermore, in these works, the role of the fillers seems to be crucial for the sound absorption properties, and not necessarily the PU network. Çelebi et al. have used leaf fibers in the preparation of composite rigid and flexible foams. Although this filler has had a major impact on the sound absorption properties of flexible foams, it had little effect in the case of rigid PUF [260].

An easy way to design a PUF with enhanced sound performance is by tuning the formulation in order to achieve the appropriate cellular structure. For example, studies have indicated the potential application of liquefied coffee grounds and CG-derived foams as sound-absorbing materials [129].

### 4.5. Other Properties and Applications

Even though PUFs have been mainly used as comfort materials and thermal/sound insulators, they can be used in other applications, but their properties need to be improved. Due to their biocompatibility, biostability, and mechanical properties, PUs can be used in biomedical applications. In fact, they were first introduced in the biomedical fields in the 1950s, when Pangman produced a composite breast prostheses covered with a polyester PUF [261]. Later, Mandarino and Salvatore used a rigid PUF for in situ bone fixation [261]. In 1960s, Biomer^®^ used an elastomeric material in cardiovascular applications [262]. Since then, PUFs have been used as central venous catheters, vascular grafts, cardiac valves, mammary prostheses, ocular implants, or drug delivery systems [261,262]. Nonetheless, in order to be used in biomedical applications, any material must present excellent biocompatibility [263]. In that sense, the biocompatibility of PUs has been extensively evaluated, both in vitro and in vivo essays [264]. However, the biostability of PUs has been an issue, because polyester-based PUs are not stable in water and in oxygen environments, and even polyether-based PUs were also not stable in vivo [261]. Yet, improvements of the biostability of PUs have been made through using more stable polysiloxanes and polyolefins [261]. The surface modification of PUs has also enlarged the use of PUFs in biomedical applications, as well as the addition of nanoparticles such as graphene, graphene oxide, and CNTs [179,265]. These improvements made PUFs one important class of materials for biomedical applications, and since then, PUFs have been used as drug release systems, scaffolds, or stents in soft tissue engineering, the absorption of biological fluids or as biocatalytic air filters, with high pH resistance, resistance to solvents, or high temperatures [179,266,267].

Nowadays, PUFs have also been used in electrical applications as conductive or insulation material [268,269]. For example, M. Argin et al. [270] studied the dielectric strength of three different foams using AC and lightning impulse voltages under different humidity and temperature conditions, and the results showed that PUFs presented two to three times better dielectric strength than air. However, for some applications, the increase of electrical conductivity is required. The growing interest in electronics, such as communication, computations, or automation increased the electromagnetic interferences (EMI), which leads to pollution. To reduce this pollution, conducting PUFs composites proved to be one of the best candidates as EMI shielding [271]. To enhance these properties, different fillers can be used. Ji Mun Kim et al. [187] studied the electrical conductivity and EMI-shielding effectiveness of rigid PUFs filled with nickel-coated carbon fiber (3.0 php), and reported that the composite showed higher electrical conductivity and electromagnetic interference-shielding effectiveness. CNTs have excellent mechanical, physical, thermal, and electrical properties, so they are excellent candidates for improving the electrical properties of PUFs. The length and aspect ratio of CNTs is an important issue that can influence the electrical properties of the materials. Jatin Sethi et al. studied the effect of multiwall CNTs morphology on the electrical and mechanical properties of PU nanocomposites, and reported that longer nanotubes exhibit better electrical conductivity, and the percolation threshold is dependent on the length and the aspect ratio [187]. The use of graphene is considered to be one of the most effective methods to increase electrical and thermal conductivity of PUFs composites. Xu Xinzhao et al. reported that the high electrical conductivity is due to the 3D networks formed by graphene on the inner surface of PUFs [272]. Similar to graphene, graphene oxide (GO) is known to be used in EMI applications [273].

However, the dispersion of these fillers in the matrix is critical, because it affects all of the properties of the foams, as discussed previously. Mercedes Santiago-Calvo et al. [274] synthetized rigid PUFs containing GO, and reported that 0.033 wt % lead to a decrease of the cell size, and consequently, the thermal conductivity. In fact, the mechanical properties were not improved using GO, because it deteriorated the cellular structure at higher GO content. Nevertheless, once these issues are overcome, due to their low price, carbon nanofillers are expected to be commonly used in the PU industry at a larger scale in the near future.

## 5. Conclusions

PUFs are a class of lightweight porous materials that have attracted enormous interest because of their wide range of properties and applications. Due to their low density, low thermal conductivity, and mechanical properties, PUFs are mainly used as thermal and sound insulators, as well as structural and comfort materials. Nonetheless, to attend to more rigorous consumers, the range of properties and applications of PUFs must be increased, which can be achieved by new process technologies or the production of PUF composites. It is also important that the production of PUFs is still highly petroleum-dependent, and in view of the ever more restrictive regulations, alternative raw materials for the production of PUFs obtained from renewable resources will have to be considered at the industrial level. New process technologies and further developments in the field of sustainability and composites will also have a major impact in the near future. Therefore, the PUFs industry must be prepared to overcome these crucial issues, and at the same time, take advantage of the potential new opportunities.
